# Case Report: Ramsay Hunt syndrome with simultaneous bilateral vestibular dysfunction as the initial manifestation in a patient with a history of breast cancer

**DOI:** 10.3389/fimmu.2025.1709001

**Published:** 2026-01-14

**Authors:** Shusheng Jiao, Miaomiao Li, Xiaofang Cheng, Liping Chen, Yan Li

**Affiliations:** Department of Neurology, Bethune International Peace Hospital, Shijiazhuang, Hebei, China

**Keywords:** bilateralvestibular dysfunction, breast cancer, peripheral facial palsy, Ramsay Hunt syndrome (RHS), Varicella-zoster virus (VZV)

## Abstract

**Background:**

Ramsay Hunt syndrome (RHS) typically presents with unilateral otalgia, herpes zoster oticus, ipsilateral peripheral facial palsy, and often ipsilateral vestibulocochlear involvement. Bilateral/contralateral vestibular dysfunction is extremely rare.

**Case presentation:**

A 60-year-old female with a history of right breast cancer presented to our clinic with 1 week of vertigo, followed by 2 days of right-sided facial deviation and otalgia. Physical examination revealed vesicular eruptions around the right ear and external auditory canal, right-sided peripheral facial palsy, spontaneous horizontal-rotatory nystagmus (with a fast phase to the left), and postural instability. Further evaluation confirmed bilateral vestibular hypofunction: the video head impulse test showed reduced gains and/or saccades in all canals; bithermal caloric testing demonstrated bilateral vestibular weakness (sum of slow-phase velocity: 10.1°/s); and symptom assessments yielded a visual analog scale (VAS) score of 7/10 and a dizziness handicap inventory (DHI) total score of 52. Facial nerve electrophysiological testing indicated significant impairment of the right facial nerve, with an amplitude reduction exceeding 50%. The stapedial reflex, Hallpike-Dix test, Roll test, pure-tone audiometry, brain and cranial nerve MRI, and routine laboratory tests showed no significant abnormalities. A diagnosis of RHS with bilateral vestibular dysfunction was established, and treatment was administered per current guidelines, including antiviral therapy, oral corticosteroids, analgesics, anti-vertigo medications, acupuncture, and vestibular rehabilitation. After 2 weeks, symptoms (facial palsy, otalgia, herpes zoster, and dizziness) improved slightly, with crusting of the herpes lesions. At the 3-month follow-up, the herpes zoster had resolved without residual pain, though mild residual dizziness (VAS 2, DHI 20) and facial weakness persisted.

**Conclusions:**

This case shows rare bilateral vestibular involvement and initial vestibular impairment preceding RHS (distinct from classical ipsilateral or rare post-RHS contralateral patterns). Elucidating the specific pathogenic mechanisms underlying this presentation holds significant clinical importance for understanding bilateral vestibular involvement.

## Introduction

Ramsay Hunt Syndrome (RHS) is an acute infectious neuropathy caused by the reactivation of varicella-zoster virus (VZV) within the geniculate ganglion of the facial nerve (1). Clinically, it is classically defined by a triad of unilateral otalgia, herpetic vesicular eruptions in the distribution of the facial nerve (e.g., around the pinna, external auditory canal, or oral mucosa) (2), and ipsilateral peripheral facial palsy—distinguishing it from Bell’s palsy, which lacks viral vesicular lesions.

Beyond facial nerve involvement, VZV reactivation often extends to adjacent cranial nerves (3), most commonly the vestibulocochlear nerve (4). This extension manifests as vestibular dysfunction (e.g., vertigo, postural instability, nystagmus) and/or cochlear symptoms (e.g., sensorineural hearing loss, tinnitus). Notably, ipsilateral vestibular impairment dominates the clinical landscape of RHS-associated vestibulocochlear involvement; existing literature indicates that the overwhelming majority of RHS cases presenting with vestibular symptoms exhibit dysfunction confined to the same side as the herpetic eruptions and facial palsy (4). Bilateral vestibular involvement (5, 6), by contrast, is exceedingly rare, with only a handful of case reports documented to date. Importantly, bilateral vestibular nerve involvement in these reported cases occurred sequentially rather than simultaneously: Teggi R et al. reported an RHS case with ipsilateral cochleovestibular hypofunction that developed acute contralateral vestibular loss 15 days after initial symptom onset (5), while Schulz P et al. described a unique case where a patient with initially right-sided RHS and complete ipsilateral vestibular loss subsequently developed left vestibular deficits 5 months later (6)—representing an unusually long interval between bilateral involvement.

Against this background, we present a rare case of RHS in a patient with a history of breast cancer and prior exposure to immunosuppressive therapy (secondary to cancer treatment). Uniquely, this patient presented with simultaneous bilateral vestibular dysfunction as the initial manifestation, which preceded the onset of typical RHS features (cutaneous herpetic eruptions and facial nerve palsy). This unusual clinical sequence—coupled with the simultaneous bilateral vestibular involvement—broadens the known clinical spectrum of RHS and highlights potential interactions between VZV reactivation and compromised host immune status, which may contribute to the development of such atypical presentations.

## Case presentation

A 60-year-old woman presented to our neurology clinic with a one-week history of vertigo, followed by the development of right-sided facial weakness and otalgia over the preceding two days. She reported no taste loss, hearing impairment, or hyperacusis. Her medical history included right breast cancer diagnosed six years ago, which was treated with breast-conserving surgery followed by adjuvant radiotherapy and chemotherapy. Owing to privacy concerns and difficulties in retrieving medical records from other healthcare facilities, the patient and her family declined to provide the relevant medical documents and specific chemotherapy regimens. Meanwhile, the patient explicitly denied a definite history of vestibular disorders (e.g. benign paroxysmal positional vertigo (BPPV), vestibular neuritis, or Meniere’ s disease), and obvious manifestations related to vestibular dysfunction (e.g. episodic vertigo, dizziness, lightheadedness, imbalance, or gait instability) over the past year. She also denied the use of common ototoxic medications, including aminoglycoside antibiotics, cisplatin, carboplatin, diuretics, benzodiazepines, and non-steroidal anti-inflammatory drugs (NSAIDs) during this period. She had no recent history of upper respiratory infection or diarrhea, and no chronic conditions such as hypertension, diabetes, hyperlipidemia, or hyperuricemia. She does not smoke and has no history of alcohol abuse.

**Figure 1:**
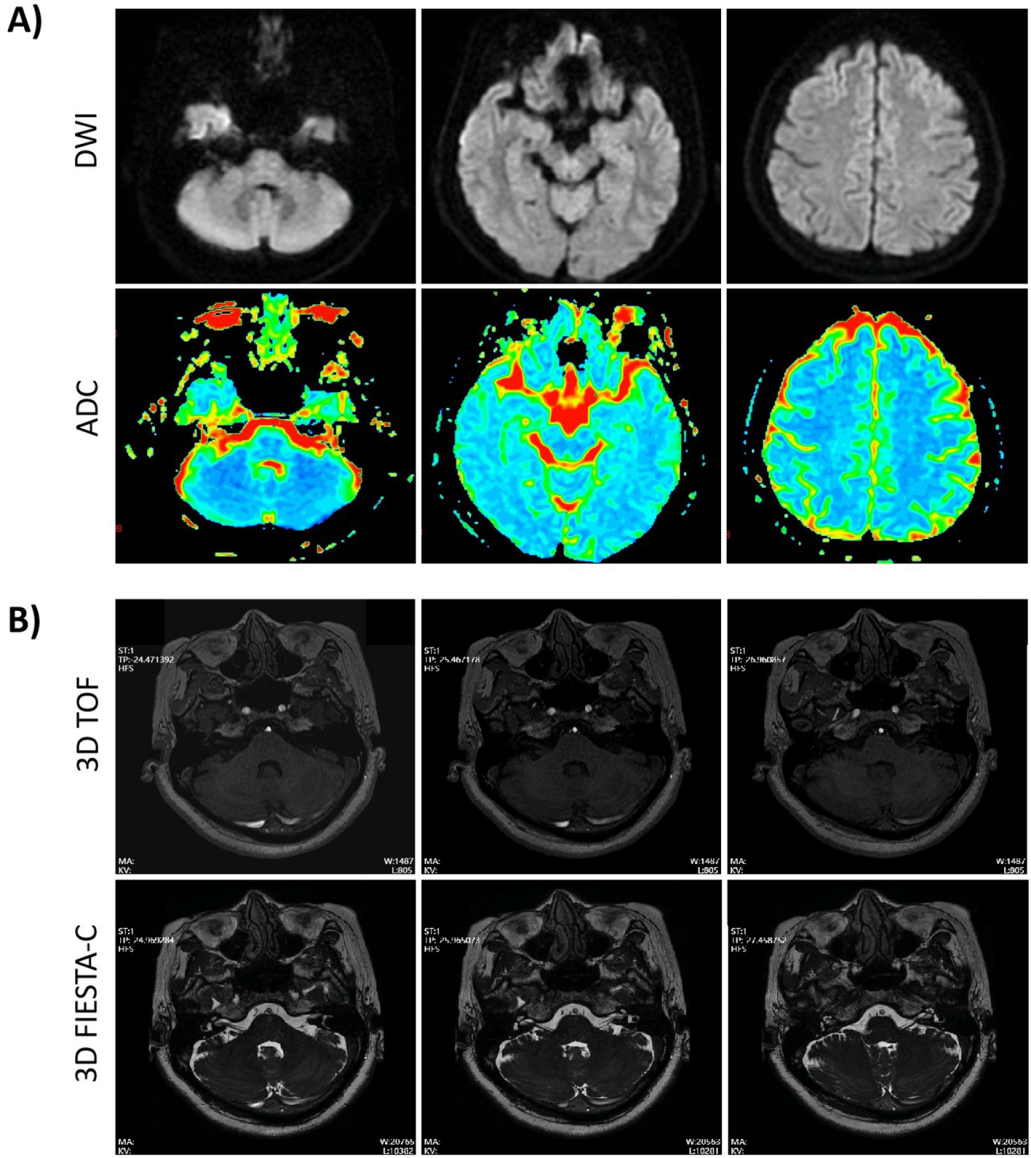
MRI findings. **(A)** Magnetic resonance diffusion-weighted imaging (MR-DWI) results obtained during the patient’s outpatient visit. No evidence of new cerebral infarction is observed (the upper row displays Diffusion-weighted imaging (DWI) sequences, and the lower row shows Apparent diffusion coefficient (ADC) maps). The three representative axial sections presented here correspond, from left to right, to the level of the internal auditory canal (including the vestibular nucleus), the cerebral peduncles, and the superior paracentral lobule. **(B)** High-resolution MRI of the facial and vestibulocochlear nerves acquired during hospitalization, showing no structural abnormalities. High-resolution images were acquired using an axial 3D FIESTA-C (Fast Imaging Employing Steady-State Acquisition with Cycled phases) sequence with a dual-excitation technique to minimize banding artifacts. MR angiography was performed using an axial three-dimensional time-of-flight (3D TOF) sequence.

**Figure 2:**
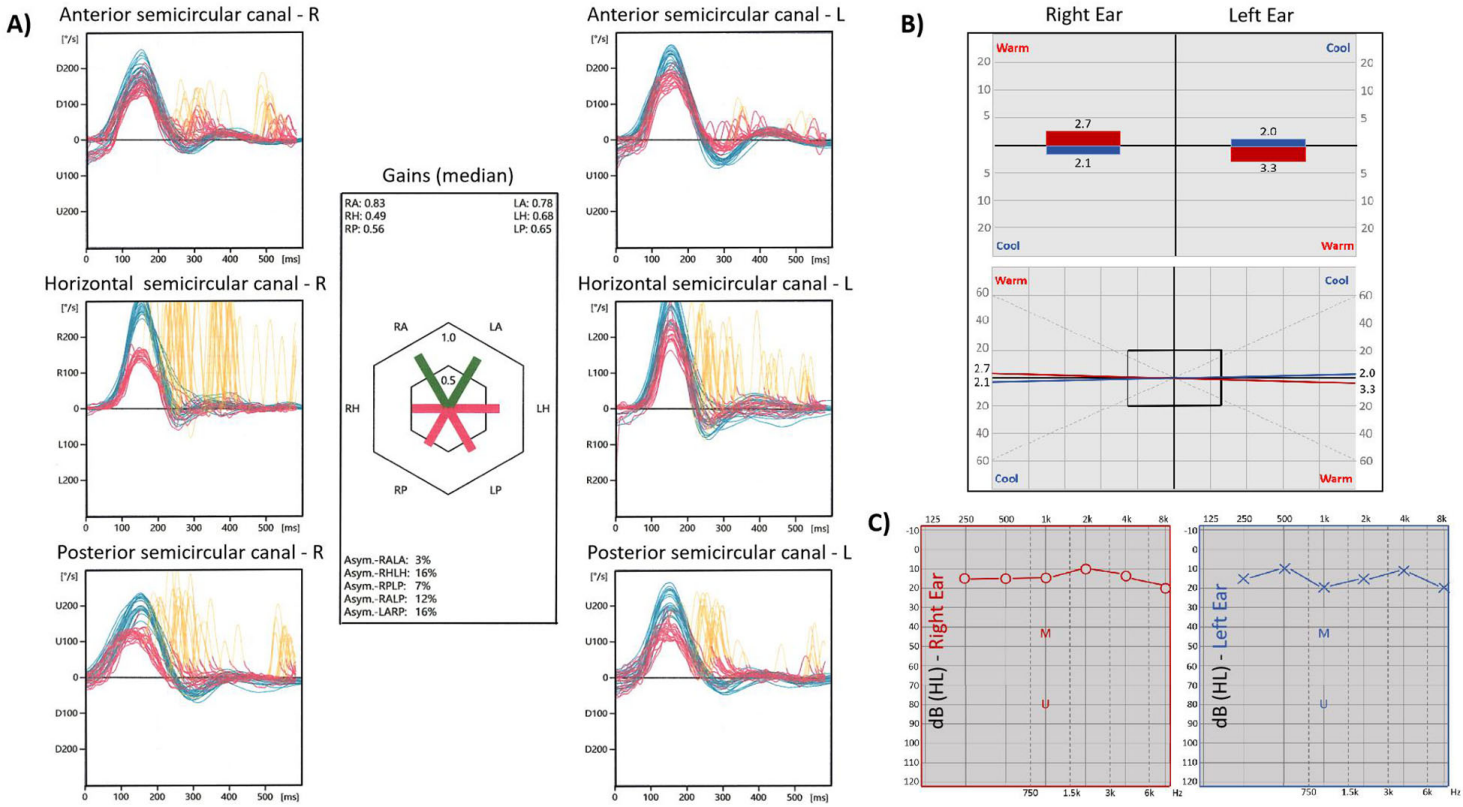
Vestibular and cochlear nerve findings pre- and post-admission. **(A)** The vHIT performed pre-admission indicated bilateral total vestibular neuritis. There is reduced gain accompanied by saccades in the bilateral horizontal and posterior semicircular canals (RH 0.49, LH 0.68; RP 0.56, LP 0.65). The right anterior semicircular canal shows normal gain (RA 0.83) with saccades, while the left anterior semicircular canal demonstrates mildly reduced gain (LA 0.78) without saccades. **(B)** The bithermal caloric test indicated bilateral vestibular hypofunction. For cool (24°C) and warm (50°C) water stimulation, the right ear showed a.SPVs of 2.1°/s and 2.7°/s, respectively, and the left ear 2.0°/s and 3.3°/s. The summed a.SPV was 4.8°/s for the right ear and 5.3°/s for the left, both below the normal threshold (≥6°/s) without significant asymmetry. **(C)** Pure-tone audiometry test showed no abnormalities. vHIT, video head impulse test; RH, right horizontal semicircular canal; LH, left horizontal semicircular canal; RP, right posterior semicircular canal; LP, left posterior semicircular canal; RA, right anterior semicircular canal; LA, left anterior semicircular canal. dB, decibel; HL, hearing level; M, masking; U, unmasking; Hz, Hertz.

During the outpatient visit, a detailed physical examination and relevant laboratory investigations were performed. General physical examination revealed normal body temperature, respiratory rate, pulse, and blood pressure. The patient was alert, cognitively intact, and cooperative throughout the assessment. Neurological examination showed vesicular eruptions around the right ear and external auditory canal, along with right peripheral facial palsy, manifested as decreased right frontal wrinkle, weakness of eye closure, deviation of the mouth to the left, inability to puff the right cheek, and flattening of the right nasolabial fold, graded as House-Brackmann Grade IV. Spontaneous horizontal-rotatory nystagmus with a fast phase to the left was observed. Postural instability was evident, including a positive Romberg test with eyes closed, as well as a wide-based gait with difficulty walking in a straight line. The Fukuda stepping test could not be adequately performed due to instability. No meningeal signs, such as neck stiffness, Kernig’s sign, or Brudzinski’s sign, were present. Motor and sensory examinations of all four limbs were unremarkable, and no pathological reflexes were elicited. Routine laboratory tests showed no significant abnormalities. Brain MRI (magnetic resonance imaging) - DWI (diffusion weighted imaging) revealed no evidence of acute cerebral infarction (**Figure 1A**). The video head impulse test (vHIT) results (detailed in **Figure 2A**) indicate bilateral vestibular hypofunction involving the afferents of all six semicircular canals to varying degrees. Specifically, reduced gain accompanied by corrective saccades was observed in the bilateral horizontal (right: 0.49, left: 0.68) and posterior canals (right: 0.56, left: 0.65). The right anterior canal showed normal gain (0.83) but with the presence of covert saccades, while the left anterior canal demonstrated mildly reduced gain (0.78) without saccades. The Hallpike-Dix test and Roll test showed no abnormalities.

Based on the aforementioned preliminary findings, the patient was diagnosed with Ramsay Hunt Syndrome accompanied by bilateral vestibular dysfunction and was subsequently hospitalized. Following admission, a series of additional examinations were performed, including facial nerve electrophysiology, stapedial reflex testing, pure-tone audiometry, bithermal caloric testing, and MRI of the facial and vestibular nerves. The bithermal caloric test revealed bilateral vestibular weakness, with a total sum of slow-phase velocity across all irrigations of 10.1°/s (right 4.8°/s and left 5.3°/s) (detailed shown in **Figure 2B**). Pure-tone audiometry results were within normal limits (**Figure 2C**). Despite preserved stapedial reflexes, electrophysiological testing revealed significant right facial nerve impairment. This was evidenced by a >50% reduction in CMAP amplitude on the right side (**Table 1**), alongside blink reflex findings of prolonged R1 and R2 latencies after right-sided stimulation with normal left-sided responses. Cranial nerve MRI, including sequences focused on the facial and vestibulocochlear nerves, showed no structural abnormalities (**Figure 1B**). Symptom burden assessment yielded a Visual Analog Scale (VAS) score of 7 and a Dizziness Handicap Inventory (DHI) total score of 52, with subscale scores of 12 (Functional, F), 22 (Physical, P), and 18 (Emotional, E). The patient declined a lumbar puncture procedure.

**Table 1:** CMAP findings of bilateral facial verves (postauricular stimulation).

Nerve/Sites	Muscle	CMAP latency (ms)	CMAP amplitude (mV)
Left facial nerve - Frontalis, Orb Oculi, Orb Oris			
Postauricular	Frontalis	3.4	1.7
Postauricular	Orb Oculi	3.6	1.4
Postauricular	Orb Oris U	4.2	1.1
Postauricular	Orb Oris L	3.3	1.9
Right facial nerve - Frontalis, Orb Oculi, Orb Oris			
Postauricular	Frontalis	3.9	0.2
Postauricular	Orb Oculi	3.9	0.2
Postauricular	Orb Oris U	4.0	0.2
Postauricular	Orb Oris L	4.1	0.1

All muscles innervated by the right facial nerve (frontalis, orbicularis oculi, superior and inferior orbicularis oris) exhibited markedly reduced amplitudes (0.1-0.2 mV, versus 1.1-1.9 mV on the left side), indicating severe functional impairment of the right facial nerve. CMAP, compound muscle action potential; Orb Oculi, Orbicularis Oculi; Orb Oris U, Orbicularis Oris Superior; Orb Oris L, Orbicularis Oris Inferior.

**Figure 3:**
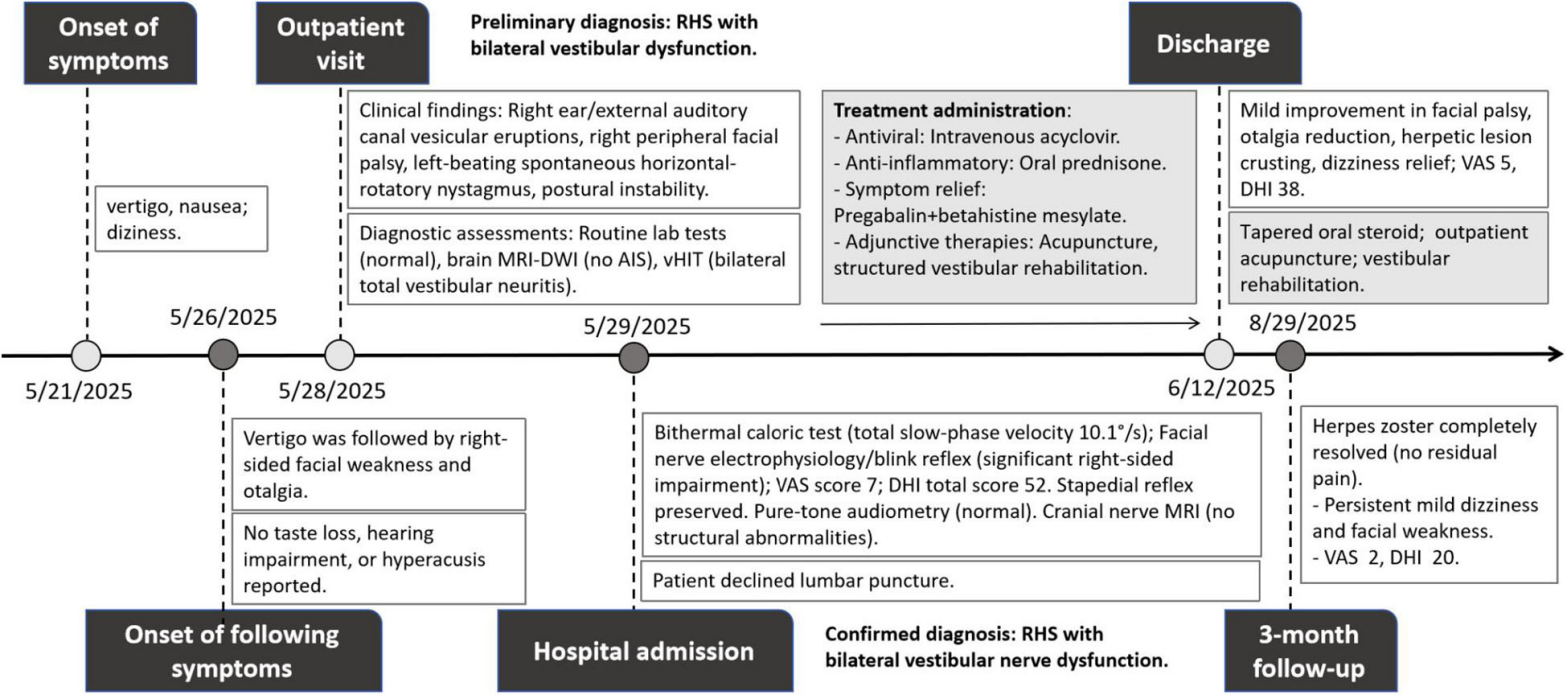
Timeline of the patient with clinical symptoms, diagnosis, and data before and after interventions. RHS, Ramsay Hunt syndrome; MRI, magnetic resonance imaging; DWI, diffusion weighted imaging; AIS, Acute ischemic stroke; VAS, visual analog scale; DHI, dizziness handicap inventory.

The aforementioned examinations further confirmed that the patient’s Ramsay Hunt Syndrome was indeed complicated by bilateral vestibular nerve dysfunction. Treatment was administered in accordance with current clinical guidelines. It included intravenous acyclovir (10 mg/kg every 8 hours), oral prednisone (1 mg/kg/day initially, tapered over 10 days), analgesics (Pregabalin 75–150 mg twice daily), and betahistine mesylate (12 mg three time daily) for vertigo. Adjunctive therapies consisted of acupuncture and structured vestibular rehabilitation. After two weeks of treatment, mild improvement was observed in facial palsy, otalgia, herpetic eruptions, and dizziness. The herpetic lesions had crusted over, and the facial nerve function improved to House-Brackmann grade III, with otalgia significantly reduced. The VAS and DHI scores decreased to 5 and 38 (Functional: 10, Physical: 16, Emotional: 12), respectively. The patient was discharged with instructions to complete the tapering course of oral steroids and to continue outpatient acupuncture and vestibular rehabilitation. At the 3-month follow-up, the herpes zoster had resolved completely without residual pain, while mild dizziness and facial weakness persisted (House-Brackmann grade II), with a VAS score of 2 and a DHI total score of 20 (Functional: 6, Physical: 8, Emotional: 6). The patient remains under ongoing follow-up. **Figure 3** illustrates the patient’s timeline, depicting the progression of clinical symptoms, diagnosis, and data collected before and after interventions.

## Discussion

RHS is pathologically defined by VZV reactivation in the facial nerve’s geniculate ganglion, with classical manifestations restricted to the unilateral distribution of the infected nerve—including otalgia, herpes zoster oticus, ipsilateral facial palsy, and ipsilateral vestibulocochlear involvement (7). The present case deviates from this paradigm in two critical ways: simultaneous bilateral vestibular hypofunction and vestibular impairment preceding classical RHS features (facial palsy and herpetic eruptions)—both of which are exceedingly rare in existing literature.

Prior reports of bilateral vestibular involvement in RHS describe sequential rather than simultaneous dysfunction. For instance, Teggi R et al. documented a case where ipsilateral cochleovestibular hypofunction was followed by contralateral vestibular loss 15 days later (5), while Schulz P et al. reported a 5-month interval between initial unilateral vestibular loss and subsequent contralateral involvement (6). By contrast, our patient exhibited bilateral vestibular hypofunction at the initial presentation—confirmed via vHIT (differential involvement of all six semicircular canals) and bithermal caloric testing (sum slow-phase velocity 10.1°/s, right 4.8°/s and left 5.3°/s, consistent with bilateral vestibular weakness). This simultaneous pattern challenges the conventional understanding of VZV’s local spread in RHS, which typically progresses unilaterally along cranial nerve branches. Equally notable is the temporal sequence of symptoms: vertigo (secondary to vestibular dysfunction) preceded facial palsy and otalgia by 1 week. Classical RHS presents with otalgia or herpetic eruptions first, followed by facial palsy and vestibular/cochlear symptoms (8). The divergent symptom chronology observed in our case suggests that bilateral vestibular dysfunction may have preceded the spread of VZV to the geniculate ganglion of the right facial nerve—a pattern seldom documented in the literature (9). Given this unusual presentation, we systematically evaluated alternative etiologies for the concurrent contralateral vestibular impairment. First, the possibility of a pre-existing occult vestibular impairment, such as bilateral vestibular neuropathy or prior subclinical contralateral vestibular neuritis, was considered, although the patient denied any preceding vestibular symptoms. Second, other undetected systemic pathologies capable of insidiously damaging bilateral vestibular structures—including autoimmune diseases, alternative infectious processes, or cerebrovascular conditions—were assessed but lacked supportive clinical evidence. Third, despite the patient’s denial of known ototoxic drug exposure, chemotherapy-induced vestibular toxicity—particularly from platinum-based agents occasionally used in breast cancer regimens—could not be definitively excluded due to the absence of detailed treatment records. Furthermore, immunosuppression secondary to the underlying malignancy and/or recent oncologic therapies may have facilitated broader neural dissemination of VZV, potentially contributing to simultaneous bilateral vestibular nerve involvement.

The patient’s medical history of right breast cancer treated with adjuvant radiotherapy and chemotherapy may serve as a key contextual factor for understanding this atypical presentation. Cancer itself, as well as its treatment modalities (e.g., chemotherapy and radiation) induce long-term immunosuppression by impairing T-cell function, B-cell activity, and innate immune responses (10–12) —critical for controlling latent VZV in sensory ganglia (including the vestibular and geniculate ganglia). In immunocompetent individuals, latent VZV is restricted to a single ganglion by local immune surveillance, explaining the unilateral nature of RHS (13). However, in immunocompromised patients, VZV may evade immune control, spread to contiguous or non-contiguous ganglia, and cause multifocal neural damage (14). In this case, immunosuppression likely enabled VZV to reactivate simultaneously in bilateral vestibular ganglia (accounting for early bilateral vestibular dysfunction) and subsequently in the right geniculate ganglion (leading to right-sided facial palsy and herpes zoster oticus). This hypothesis aligns with prior reports linking immunosuppression (e.g., AIDS, organ transplantation, cancer) to atypical VZV manifestations, such as multifocal zoster or bilateral cranial nerve involvement (15–18). While we cannot definitively confirm VZV in vestibular ganglia (given the lack of lumbar puncture or ganglion biopsy), the temporal association between immunosuppression, atypical vestibular findings, and classical RHS features strongly supports this mechanism. Notably, the patient’s preserved hearing (normal pure-tone audiometry) and stapedial reflex suggest selective VZV involvement of vestibular ganglia rather than cochlear structures—a further distinction from typical RHS, where vestibular and cochlear dysfunction often coexist (1). This selectivity may reflect differential vulnerability of vestibular neurons to VZV in the setting of immunosuppression, though the exact reason remains unclear and warrants further investigation.

We hypothesize that the pathogenic mechanisms underlying RHS complicated by bilateral vestibular nerve impairment may primarily revolve around VZV dissemination and host immune responses—though this remains a tentative supposition, given the extreme rarity of such cases and the limited clinical evidence currently available to fully validate this framework. First, in immunocompromised patients, VZV may spread either contiguously between bilateral vestibular ganglia via the subarachnoid space or hematogenously to infect the contralateral vestibular ganglion, directly damaging vestibular neurons and their axons (the vestibular nerves) and disrupting their normal function (19). Second, immune-mediated bilateral neuroinflammation plays a critical role: after VZV reactivation, the host’s immune response (including T-cell infiltration and proinflammatory cytokine release) targets infected neural tissues. This inflammatory cascade can extend beyond the ipsilateral geniculate ganglion to involve both vestibular nerves, even in the absence of overt contralateral VZV replication, leading to “bystander” immune damage that impairs bilateral vestibular function (20). Third, cross-ganglionic spread via interconnected neural networks facilitates bilateral involvement: VZV may traverse neural pathways linking the vestibular ganglia to brainstem vestibular nuclei, then spread retrogradely or anterogradely to reach the contralateral vestibular system. This spread is particularly likely in immunocompromised patients, whose impaired viral clearance capacity fails to contain VZV, allowing it to disrupt bilateral vestibular nerve function (3). Although bilateral symmetric cutaneous involvement in herpes zoster is exceptionally rare, bilateral neurological complications from VZV are well-documented in the literature, particularly in the context of immunosuppression or vasculopathy. Reported manifestations include bilateral acute retinal necrosis (21, 22), optic neuritis (17), encephalitis localized to bilateral temporal lobes (23), and even bilateral facial palsy (16). Notably, the recent case of bilateral facial palsy due to zoster sine herpete (VZV reactivation without rash) (16) provides a direct precedent for simultaneous bilateral cranial nerve involvement in the absence of bilateral skin eruptions (“zoster sine herpete”). This supports the biological plausibility of our hypothesis.

This case also carries important clinical implications for the diagnosis and management of RHS and bilateral vestibular dysfunction. It expands the differential diagnosis of bilateral vestibular hypofunction, which is most commonly attributed to autoimmune inner ear disease, ototoxicity, or neurodegenerative disorders (24). However, our findings indicate that RHS—especially in immunocompromised patients—should be added to this differential list, even in the absence of classical RHS features such as facial palsy or herpetic eruptions. The patient’s partial response to treatment further underscores the value of early antiviral and steroid therapy: intravenous acyclovir and oral prednisone led to improvements in facial palsy (from House-Brackmann Grade IV to II), reduced dizziness, and complete resolution of herpes zoster at the 3-month follow-up, which aligns with current clinical guidelines recommending early antiviral and anti-inflammatory therapy for RHS (25). For patients with concurrent vestibular dysfunction, adjunctive treatments such as vestibular rehabilitation and anti-vertigo medications (e.g., betahistine) may further enhance functional outcomes, as demonstrated by the reduction in the patient’s DHI score from 52 to 20 at the 3-month follow-up. Additionally, the persistence of mild residual dizziness and facial weakness at the 3-month follow-up is consistent with prior reports indicating that vestibular recovery in RHS may be incomplete, particularly in cases of severe or bilateral vestibular involvement (26). This highlights the need for prolonged outpatient follow-up and continued rehabilitation to optimize the patient’s quality of life.

This case has several limitations. First, we did not perform a lumbar puncture to detect VZV DNA in the cerebrospinal fluid or a vestibular ganglion biopsy—both of which are considered the gold standards for confirming VZV involvement of vestibular structures (25). Additionally, we did not conduct contrast-enhanced MRI of the facial and vestibular nerves; this lack of imaging precluded us from obtaining radiological evidence of bilateral vestibular nerve involvement, as well as evidence of vascular loop compression on the vestibular or facial nerves, which may have introduced bias into the analysis of the underlying etiology. Second, we lacked longitudinal vestibular function testing (e.g., repeated vHIT or bithermal caloric testing) to assess the rate and extent of vestibular recovery. Furthermore, systematic vestibular-cochlear nerve function assessments—such as ocular and cervical vestibular myogenic potentials (oVEMP and cVEMP)—were not performed; this omission significantly hindered our ability to accurately determine the degree of involvement of the vestibular and cochlear nerves. Third, the patient’s prior chemotherapy regimen was not documented in detail, precluding a thorough analysis of the role of specific chemotherapeutic agents in inducing immunosuppression as well as potential vestibular dysfunction. Fourth, the coexistence of multiple cranial nerve involvement necessitates consideration of alternative etiologies such as Guillain-Barré syndrome and paraneoplastic syndrome. However, the patient declined relevant diagnostic evaluations for these conditions, which further increased uncertainty in the clinical differential diagnosis.

## Conclusion

This case documents an extremely rare presentation of RHS characterized by bilateral vestibular hypofunction as the initial manifestation, which deviates from the classical pattern of unilateral vestibulocochlear involvement in typical RHS and even the rare post-RHS contralateral vestibular dysfunction. Elucidating the specific pathogenic mechanisms underlying this presentation holds significant clinical importance for understanding bilateral vestibular involvement. While prior oncological treatment represents a potential contributor to altered immune status that may have facilitated atypical VZV dissemination, this mechanistic link remains an exploratory hypothesis, given the lack of detailed treatment exposure data and the patient’s cancer remission status.

## Data Availability

The datasets presented in this article are not readily available because of ethical and privacy restrictions. Requests to access the datasets should be directed to the corresponding authors.
